# Stephen Jay Gould’s Analysis of the Army Beta Test in The Mismeasure of Man: Distortions and Misconceptions Regarding a Pioneering Mental Test

**DOI:** 10.3390/jintelligence7010006

**Published:** 2019-02-20

**Authors:** Russell T. Warne, Jared Z. Burton, Aisa Gibbons, Daniel A. Melendez

**Affiliations:** 1 Department of Behavioral Science, Utah Valley University, Orem, UT 84058, USA; jburton@uvu.edu (J.Z.B.); aisagibbons@gmail.com (A.G.); 2 Department of Counseling Psychology, University of Denver, Denver, CO 80210, USA; daniel.melendez@du.edu

**Keywords:** intelligence, history of psychology, intelligence testing, Army Beta, Stephen Jay Gould, *The Mismeasure of Man*

## Abstract

In *The Mismeasure of Man*, Stephen Jay Gould argued that the preconceived beliefs and biases of scientists influence their methods and conclusions. To show the potential consequences of this, Gould used examples from the early days of psychometrics and allied fields, arguing that inappropriate assumptions and an elitist desire to rank individuals and/or groups produced incorrect results. In this article, we investigate a section of *The Mismeasure of Man* in which Gould evaluated the Army Beta intelligence test for illiterate American draftees in World War I. We evaluated Gould’s arguments that the Army Beta (a) had inappropriate content, (b) had unsuitable administration conditions, (c) suffered from short time limits, and (d) could not have measured intelligence. By consulting the historical record and conducting a pre-registered replication of Gould’s administration of the test to a sample of college students, we show that Gould mischaracterized the Army Beta in a number of ways. Instead, the Army Beta was a well-designed test by the standards of the time, and all evidence indicates that it measured intelligence a century ago and can, to some extent, do so today.

## 1. Introduction

Stephen Jay Gould’s *The Mismeasure of Man* [1] was a popular science book of the late twentieth century. In this work, Gould argued that social scientists are often blinded by their preconceived views. This bias often results in faulty measurement methods, distorted data, and incorrect conclusions. Gould was especially concerned about how intelligence research and IQ testing could have harmful consequences for individuals. He saw the early pioneers of psychometrics as being grounded in racist assumptions about the relative intelligence level of different groups, and that these scientists’ results reinforced and justified their political and social views. Gould believed that these assumptions ultimately culminated in policies that would harm disadvantaged individuals and groups.

Gould’s book won awards and was well received in the popular press and among non-experts (e.g., [2]). However, in the scholarly press, the reception was much colder [3]. For example, Jensen [4] criticized many aspects of Gould’s book, including a focus on obsolete examples from decades earlier, misrepresenting Jensen’s views, and what Jensen claimed was Gould’s penchant for discussing “… a museum collection of scientific fossils and … many a straw person of his own making” ([4], p. 124). Snyderman and Herrnstein [5] argued that Gould misstated the historical record about the relationship between intelligence tests and American restrictions on immigration in the early 1920s. Carroll [3] detailed the many technical and philosophical errors in Gould’s explanation of factor analysis. Despite these criticisms, Gould’s book remains popular. According to Google Scholar, the book was cited 445 times in 2017 alone and over 10,000 times since its publication. 

One common criticism (e.g., [6]) of Gould’s book is that he fell prey to many of the scientific sins he accused early psychometricians of. Reviewers see Gould’s leftist politics and social views as the fundamental basis of his book [2], and Gould ([7], pp. 36–39) openly acknowledged in the revised version that he was motivated to write the book by his political and social views:

My original reasons for writing *The Mismeasure of Man* mixed the personal with the professional. I confess, first of all, to strong feelings on this particular issue. I grew up in a family with a tradition of participation in campaigns for social justice, and I was active, as a student, in the civil rights movement at a time of great excitement and success in the early 1960s… Some readers may regard this confessional as a sure sign of too much feeling to write a proper work in nonaction. But I am willing to bet that passion must be the central ingredient needed to lift such books above the ordinary, and that most works of nonfiction regarded by our culture as classical or enduring are centered in their author’s deep beliefs.

([7], pp. 36, 39).^1^

Critics claimed that these views influenced Gould’s conclusions (e.g., [4,8,9,10]). 

One interesting recent development is the possibility that Gould himself distorted the data that he reported in his book. When Gould evaluated Samuel George Morton’s work on cranium size differences among racial groups, Gould claimed Morton selectively reported, manipulated, and distorted data in order to reach a conclusion about the supposed racial superiority of Europeans (on the basis of a larger skull capacity). Yet, Lewis et al. [11] argued that Gould himself selectively reported, manipulated, and distorted data in order to bolster his argument that Morton worked to support his preconceived notions. Lewis et al.’s [11] claim was contested by Weisberg [12], who argued that Lewis et al.’s [11] re-analysis was flawed, and that Gould’s arguments were mostly sound. However, the incompleteness of Morton’s reporting and problems with his sampling led Kaplan, Pigliucci, and Banta [13] to argue that Morton’s analysis was indeed flawed, but that Gould was equally mistaken in believing that any re-analysis would be useful in understanding Morton’s motives and actions. The debate continues, with a new article [14] indicating that Morton’s interpretations were racist, but that his data collection was accurate. According to Mitchell, Gould was correct that Morton had preconceived viewpoints that he tried to use science to justify, but Gould was wrong about the accuracy of Morton’s data. Mitchell believes that Gould’s fanciful story of Morton consciously or unconsciously influencing the data during the measurement process is not supported by the data.

Regardless of one’s position about whether Morton was honest in his data collection and analysis or whether Gould was justified and/or correct in his assessment of Morton’s work, the criticisms of Gould—even by his supporters—(e.g., [12], pp. 173, 175) suggest that the morality tale of Morton as a biased scientist attempting to support his racist views is (at the very least) simplistic. This episode also indicates that other sections of *The Mismeasure of Man* could bear re-examination. The current study does so by giving a careful assessment to one section of *The Mismeasure of Man*: the examination of the Army Beta intelligence test ([1], pp. 199–222),^2^ a section of Gould’s book never before subjected to informed scrutiny.

## 2. Background of the Army Beta

Historical data regarding the United States army’s testing program during World War I is taken primarily from a book edited by Robert Yerkes [15], which had as its primary authors Lewis Terman, Edwin Boring, and Harold Bingham. However, other individuals—often uncredited—involved with the army testing program also contributed to the book ([15], p. vi). A second resource is another edited volume by Yoakum and Yerkes [16], which was prepared by Terman, Mabel Fernald, Raymond Dodge, and other uncredited individuals. This latter book consisted of material that was a less technical summary of the most important aspects of the Yerkes [15] volume. Readers should be aware that both books were a committee effort and that we will use a shorthand of referring to specific passages as being authored by “Yerkes” or “Yoakum and Yerkes,” even though Yerkes may not be responsible for writing the passage in question, and it is not clear how much Yerkes personally approved of the text. Gould [1] usually followed this convention (though he ascribed all analyses to Boring, which may or may not be historically accurate). Our citing of these texts in this manner seems appropriate as long as one recognizes that sometimes the moniker “Yerkes” applies to a group of authors or an unknown author, and not necessarily Robert Yerkes himself. (This is not unlike classicists who refer to the authors of *The Iliad* and *The Odyssey* as “Homer,” though most agree that these works do not have just one author.) Robert Yerkes was the sole author or a co-author of three minor articles related to the army intelligence tests [17,18,19], though none of these provided unique information about the Army Beta. After the war, Robert Yerkes resumed his research into animal cognition, and his interests turned to almost exclusively to research on primates [20]. He published nothing more about psychological testing in humans after 1923.

The Army Beta was one of two group intelligence tests administered to American men drafted into the army during World War I. It was designed for men who could not read and/or speak English fluently, whereas its companion test, the Army Alpha, was designed for men who were fully proficient in written and spoken English [16]. Literacy was determined in different ways at different army camps, but the most typical method was by asking the men if they could (a) read and understand a newspaper and (b) write a letter home. Some men also had to have a certain number of years of schooling to be considered literate, though the minimum requirement varied across testing locations (see [15], p. 744). Men who did not meet these requirements took the Army Beta. Some men took both tests because it was found after the Army Alpha that they spoke and/or wrote English “with difficulty” or scored extremely poorly on the Army Alpha ([15], pp. 100, 156; [16], pp. 51–52), the latter being approximately 8% of Army Alpha examinees. About 30% of all tested draftees in 1918 took the Army Beta ([16], p. 9), of whom about 20% also took the Army Alpha ([15], p. 100). Between April 28, 1918, and January 31, 1919, a total of 483,469 men took the Army Beta (See [21,22]] for an accessible description of the creation and implementation process for the army tests.)

The Army Beta consisted of seven subtests. The first (“Maze”) was a series of five mazes that examinees had to trace a path through. The second subtest was called “Cube Analysis” and consisted of 16 two-dimensional drawings in which an examinee must determine the number of cubes in the picture, even though some cubes are partially or completely obscured. The third subtest was called “X-O Series” and required examinees to complete twelve patterns of X’s and O’s. The “Digit Symbol” subtest was the fourth subtest and required examinees to use a key at the top of the page to replace a series of numbers with the correct symbols. (This subtest greatly resembles coding subtests on modern intelligence tests.) The fifth subtest on the Army Beta was “Number Checking” and required examinees to mark whether two series of numbers were different. Subtest 6 (called “Picture Completion”) consisted of 20 drawings, each missing an important component of the image, which examinees must then draw to complete the picture. Finally, the last subtest was called “Geometric Completion” and required the examinee to complete a diagram showing how two or three given shapes can be combined to form a square. Time limits on each section ranged from 1 minute 45 seconds to 3 minutes for each subtest. Scores from each subtest were summed and then converted to a rating that could range from A (the highest rating) to D- (the lowest rating).

Ratings on the Army Alpha and Army Beta were reported to commanding officers, but officers’ use of these scores varied. Some ignored the scores completely, while others attempted to balance out military units so that no unit had a preponderance of men who scored high or low on the tests [15,21]. About 5% of men scored very poorly on the Army Alpha and/or Army Beta, and these soldiers received a standardized individual intelligence test, such as the Stanford-Binet. After individual testing, most men were assigned to regular army duty, but a minority experienced other outcomes. Out of all examinees, 0.50% were discharged, 0.64% were assigned to service organizations (which principally engaged in manual labor), and 0.61% were assigned to development battalions, which were military units designed to make men with moderate mental or physical deficiencies fit for regular duty ([15], p. 101). For most men, however, their Army Alpha and/or Army Beta scores had little to no noticeable effect on their military career [21]. No man was eliminated from military service solely on the basis of his intelligence test scores [15,16,21], nor were people promoted just because they had scored highly on an intelligence test, though sometimes a low score could prevent men from being promoted [23].

## 3. Gould’s Criticisms of the Army Beta

Most of Gould’s criticism of the Army Beta falls into four categories, which are criticisms of (a) test content, (b) test administration conditions, (c) the Army Beta subtests’ time limits, and (d) the claim that the Army Beta could measure intelligence at all—especially for illiterate or non-English speaking examinees. Each of these criticisms will be recapitulated and investigated in light of the historical evidence about the Army Alpha and Army Beta, most of which is taken from the official reports about the tests’ development, administration, and results [15,16]. For criticisms (c) and (d), we will also report our experience replicating Gould’s administration of the Army Beta to college students.

### 3.1. Criticisms of Test Content

The degree to which items on a psychometric test appear to measure what a test’s creators claim the items measure is called *face validity* [24]. Gould examined Army Beta content and found it wanting. He claimed, for example, that the picture completion test contained “blatant cultural biases” ([1], p. 212), but never explained which items would be culturally problematic. He merely wrote, “Best of luck with pig tails, crab legs, bowling balls, tennis nets, and Jack’s missing diamond [on a playing card], not to mention the phonograph horn (a real stumper for my students)”^3^ ([1], p. 210). Though Gould may have seen the difficulties of these items as obvious, he never explains *why* illiterate individuals or non-English speaking immigrants would have difficulty with these items. In fact, it is easy to imagine why some of these items could actually be easy to illiterate or foreign-born examinees. For example, in the United States at the time, rural residents were twice as likely to be illiterate, compared to urban residents ([25], p. 1207). It is not unreasonable to assume that rural illiterate men taking the Army Beta had been previously exposed to pigs and that the flaw of an image of a pig without a tail would be easy to discern for an illiterate examinee.

This demonstrates the problem of face validity judgements: they are entirely subjective. Moreover, they are fallible because item surface content often does not reflect the actual mental processes that test items measure. Face validity judgments provide very little information about actual test functioning, as testing experts have known for decades (e.g., [26]; [27], p. 304; [28], pp. 39, 76–77, 114–115; [29], p. 135). This is a particular problem with intelligence tests, because of “the indifference of the indicator” ([30], pp. 197–198). This principle is that any test item measures intelligence to at least some degree—as long as responding to the item correctly requires some sort of cognitive effort [31].

Gould’s ([1], p. 212) other criticism of the Army Beta test content was the test’s use of written numbers. The Cube Analysis subtest requires examinees to write numbers, and the Digit Symbol and Number Checking subtests both use numbers in their stimuli. Yet, the use of numbers was likely not a serious impediment for most examinees. The majority of Army Beta examinees were not immigrants—approximately 60–65% by our estimate using Yerkes’s [15] data—and likely spoke English with native fluency. Many Army Beta examinees had attended at least some school, and about 20% originally qualified for the Army Alpha. Even among the foreign-born examinees, the use of written numbers was likely not problematic because almost 70% of foreign-born men drafted in World War I had lived in the United States for over 5 years, and over 30% had lived in the country for more than a decade ([15], p. 704). Indeed, one-third of foreign-born army draftees were proficient enough in English that they could take the Army Alpha ([15], p. 696). Recent immigrants (who had been in the United States for less than 5 years) were only 10-12% of examinees, and it is likely that some of these men spoke English.^4^ This contradicts Gould’s ([1], p. 206) statement that, “Many Beta examinees were recent immigrants who did not speak English.”

Gould’s face validity argument against the Army Beta requires readers to believe that large numbers of Army Beta examinees were completely baffled by the use of numbers on the test, despite these being some of the most basic written symbols in the English language—and almost any language that the examinees spoke as a native. With hundreds of thousands of men taking the Army Beta, it is likely that some men were genuinely confused about the test, but the available evidence appears to suggest that Gould’s claims regarding numerical comprehension were exaggerated.

### 3.2. Criticisms of Test Administration Conditions

#### 3.2.1. Army Beta Instructions

Much of Gould’s [1] discussion of the Army Beta is occupied with dissecting the instructions to the examinees for the Army Beta. Gould [1] claims that the conditions of test administration were “Draconian” (p. 205) and “diabolical” (p. 206). As a result, “… most of the men must have ended up either utterly confused or scared shitless” ([1], p. 205). Gould had no way to know if “most of” the men were scared, considering no data were recorded of the men’s feelings. Also, Gould’s conclusion of the fear and anxiety of examinees is based almost entirely on his reading of the instructions and his conjecture of what examinees’ reactions “must have” been. Yet, this is not the only interpretation of the instructions. Carson [21], for example, saw the instructions on both the Army Alpha and Army Beta as an effort to make the tests fit in with the military setting and to distance intelligence testing from its academic origins.

Apart from his subjective opinion of what a soldier’s reaction to the Army Beta instructions would have been, the only other source Gould had for this judgment was an article from Kevles [23], who quoted a letter from an Army Beta examining officer written over 40 years after the army testing program was abolished. This examiner stated, “It was touching to see the intense effort … put into answering the questions, often by men who never before had held a pencil in their hands” ([23], p. 576). Needless to say, there is a vast difference between struggling and being “scared shitless.”

Gould [1] also criticized the Army Beta instructions because—in contrast to Army Alpha examinees—soldiers taking the Army Beta test were given little information about the purpose of the test. This is a fair criticism. Army Alpha examinees were told, for example, that they were not expected to get a perfect score on the test, that the test would be used in determining personnel assignments, and that commanding officers would have access to the scores. However, Army Beta examinees only received instructions on what to do to complete each subtest on the exam. Gould ([1], pp. 205–206) believed that this lack of information increased the anxiety of examinees, though there is nothing in the historical record to indicate this. Moreover, it is not clear how one additional paragraph of information would give an advantage to Army Alpha examinees (who, one must keep in mind, were also about 20% of the Army Beta examinees). In fact, one could imagine that learning that one’s commanding officer would have access to scores describing how one “… can remember, think, and carry out what you are told to do” ([15], p. 157) could increase Army Alpha examinees’ anxiety. Once again, Gould’s entire argument against this aspect of the Army Beta was based on his speculation of how examinees would react to the testing environment.

Gould also mischaracterized the method of test administration for the Army Beta, which relied mostly on pantomime and simple commands. Gould asserted that the use of this method was due to “… Yerkes’s poor opinion of what Beta recruits might understand by virtue of their stupidity” ([1], p. 206). There is nothing in the historical record to support this statement. Yerkes and his fellow test creators did not consider Army Beta examinees inherently stupid. In fact, the entire purpose of the Army Beta’s creation was “… to give to those who have made a low score in group examination *a* [the preliminary version of the Army Alpha], a more favorable chance to show what they can do” ([15], p. 128, see also p. 368]. Indeed, men who failed the Army Alpha and then took the Army Beta often performed much better on the latter test ([15], p. 380). The minimal instructions seem to be part of the creators’ efforts to design a test that would be accessible to as broad a population of illiterate men as possible ([15], p. 368).

Despite the simple instructions and example items that Army Beta examinees received, Gould ([1], p. 212) claimed many men were “… completely befuddled.” Yet, this seems unlikely because the subtests—and the accompanying instructions—had been piloted multiple times at military training camps with soldiers and draftees of various education and English proficiency levels to ensure that examinees understood what to do (see [15], Part II, Chapters 6 & 7). Gould also neglected to mention that during the test proctors could also give brief instructions and feedback in foreign languages; Russian and Italian—two of the most common native language for immigrants at the time—were specifically mentioned and others were permitted ([15], p. 164).

The only empirical evidence Gould presented to support his claim that many examinees did not understand what to do was based on the presence of a large number of zero scores on some subtests, indicating that some men did not answer a single item on a subtest correctly. Gould interprets this as indicating that the men “… could not fathom the instructions …” ([1], p. 213). However, Gould overstated the frequency of zero scores on the Army Beta subtests.^5^ Based on the histograms that Yerkes ([15], pp. 627–628) reported (some of which Gould reproduced in *The Mismeasure of Man*), the percentage of examinees who scored zero on a subtest was never very large: ranging from about 0.6% for the Picture Completion subtest to about 9.7% for the Geometric Completion subtest, with an average percentage of zero scores on the Army Beta subtests of about 5.1%. These figures, however, were derived from a “select group” who were also literate enough to take the Army Alpha. Percentages of zero scores on each subtest in a more representative sample are not reported, though. Overall, only 4.0% of examinees earned a total score less than 10 on the Army Beta, and only 2.6% scored less than 5 ([15], p. 669). Thus, it is likely that most men who took the Army Beta knew what to do on at least some subtests—a fact that contradicts Gould’s ([1], p. 217) claim that “vast numbers of men” earned zero scores, and therefore, must not have been able to understand the Army Beta test instructions and/or stimuli. Gould also argued ([1], pp. 214–217) that men with scores of zero were penalized by having their scores statistically adjusted to be a negative value. However, this adjustment was only for the purposes of producing tables that equate Army Alpha, Army Beta, and individually-administered intelligence test scores. No men were actually given negative scores or individually judged as being “… too stupid to do any items,” or “dullards” ([1], p. 216).

But Gould did not suggest another possibility: perhaps some subtests were more difficult than others, and that more difficult tests would result in a larger number of zero scores than other subtests. In fact, pilot data ([15], pp. 375–377) from the Army Beta’s development indicates that the subtests Gould believed had unclear instructions were indeed the hardest subtests. Ironically, some subtests were eliminated from the Army Beta in its development stage because they produced too many zero scores ([15], pp. 372–377). This indicates that the Army Beta’s creators were aware of the problem of an excess of zero scores and took efforts to mitigate it. Still, subtests with a meaningful proportion of zero scores would be problematic because they produce scores that are ambiguous in meaning. The Army Beta’s creators not only recognized that a zero score on an intelligence test may indicate that the examinee was extremely low scoring or did not understand the instructions, but that—based on the test alone—it was impossible to tell why an examinee scored so poorly ([15], p. 330). This is why examinees who performed extremely poorly on the Army Beta were then examined individually and men were not rejected from military service solely on the basis of a poor Army Beta score.

Further evidence that non-English speakers understood the instructions of the Army Beta comes in a comparison of scores from draftees who had a fourth grade education (the lowest level of education that data were reported for). For both the Army Alpha and Army Beta examinations, foreign-born White draftees scored slightly *higher* than native-born White draftees ([15], p. 773), thus indicating that any potential language barrier was likely not a problem for the least educated examinees. Even for a relatively more educated group (with an eighth grade education), the native-born White draftees had a median score only 1.9 points (out of 118) higher on the Army Beta than the median score for foreign-born White draftees ([15], p. 776), a difference that we estimate is equivalent to *d* = 0.088 (based on the data in ([15], p. 669). A 1.9 point disadvantage for foreign-born White examinees was too small to impact the typical group member’s classification.

#### 3.2.2. Test Administration Facilities

In addition to the test instructions, Gould was critical of the conditions under which the test was administered. Judged by modern American standards—in which cognitive tests are usually administered in a quiet, comfortable room with few distractions—Gould is correct that the army conditions were “… something of a shambles …” ([1], p. 201). The lack of pre-existing infrastructure for large-scale testing and the apathy or antagonism of some commanding officers meant that often examiners at military training camps had to “make do” with the facilities that were available to them. At many camps, examination buildings were used for multiple purposes or were reappropriated for psychological testing. Some men took their Army Alpha or Army Beta tests in storage buildings, hospital buildings, mess halls, and auditoriums ([15], pp. 69–87). Adequate furnishings were not always available, and some examinees took their test while seated on the floor (see images between pp. 90 and 91 of [15]) because separate examining buildings with adequate seating were not available. Much of the first part of the Yerkes [15] book describes the difficulties the army psychologists faced in securing ideal facilities for the army testing program, though facilities and procedures seemed to have improved as World War I progressed. 

As a result of the less than ideal testing facilities, Gould claimed that often the facilities had “… inadequate acoustics, illumination, and lines of sight” ([1], p. 201). There is nothing in Yerkes’s [15] book to support the claim that examinees “often” had trouble seeing or hearing examiners because of room space. Instead, the most common complaint about the facilities in the [15] report is that the size of the facilities and number of personnel were inadequate for doing all the desired testing. Some men who scored low on the Army Alpha were unable to take the Army Beta instead, and many who failed to pass the Army Beta were not examined individually by a psychologist. Given the lack of testing infrastructure, the time required to train psychologists, and the sheer number of men passing through the training camps, these inadequacies are not surprising.

Gould (e.g., [1], p. 201) also quoted a report from Camp Hancock, Georgia (originally printed in ([15], pp. 105–108), that stated that the intelligence ratings derived from the tests scores were not trustworthy: “Part of this inaccuracy I believe to be due to the fact that the room in which the examination is held is filled too full of men. As a result, the men who are sitting in the rear of the room are unable to hear clearly and thoroughly enough to understand the instructions.” Gould claimed that this statement originated from the “chief tester” at the camp, but this is not true. The letter was written by the commander of Camp Hancock, who quoted the opinions of fourteen commanding officers at the camp; the quote that Gould reproduces in his book was the only unfavorable view and was from “Commanding Officer, Group No. 5,” *not the chief tester*. (In fact, none of the 14 reports are from officers who conducted psychological testing.) Gould also chose to quote the *only* unfavorable report from the 14 officers at Camp Hancock. *All* the commanding officers of training camps (all of whom were non-psychologists who had no vested interest in the success of the military testing programs) thought that the results were valuable for their work ([15], pp. 104–114), despite any unfavorable conditions that may have prevailed at the camps under their command. If Gould is correct and conditions were so unfavorable that they invalidated the intelligence test scores, very few army personnel alive at the time seemed to have noticed.

#### 3.2.3. Screening for Literacy

A final test administration procedure that Gould criticized was the nonstandardized methods of determining literacy of recruits in order to determine which soldiers should take the Army Alpha and which were eligible for the Army Beta. Gould claimed that these irregularities “… deprived the summary statistics of any meaning” ([1], p. 202). While Gould is correct that the variability in sampling examinees makes some score comparisons (e.g., across camps or across Alpha and Beta) shaky at best, it did not invalidate the interpretation of the meaning of the scores for most individual examinees. Such irregular selection for examination is common even today. For example, within the United States, the percentage of high school students who take the ACT or SAT college admissions tests varies greatly from state to state. While this inconsistency in examinee selection is inadequate for making comparisons across state lines, it does not invalidate these tests as measures of college preparedness. Likewise, the inconsistent methods for screening soldiers for the Army Alpha or Army Beta did not invalidate these tests as measures of intelligence for most examinees.

### 3.3. Criticisms of Time Limits

Gould was also highly critical of the time limits of the Army Beta subtests, stating that it “… was virtually impossible to finish at least three of the tests …” ([1], p. 206) and that “… the examination was conducted in an almost frantic rush” ([1], p. 210). He was particularly critical of the time limit for the X-O Series (with a time limit of 1 minute 45 seconds to complete 12 patterns by filling in 4-10 squares each), Digit Symbol (which had a time limit of 2 minutes to write 90 symbols in 6 sets), and Number Checking (which required examinees to compare 50 pairs of numbers, each consisting of up to 11 digits, in 3 minutes). Gould criticized these subtests’ short time limits because of his experience administering the Army Beta to 53 undergraduates in his course on “biology as a social weapon” at Harvard University. In each of these subtests, a majority of Gould’s students could not finish every test item—a fact which seems to be the entire basis for his criticism of these subtests. (Table 1 displays the number of Gould’s students who completed each subtest).

However, the army psychologists chose these time limits after piloting longer time limits with hundreds of examinees. During the Army Beta’s development stage, time limits for the subtests ranged from 2 to 5 minutes, with each test having a time limit that ranged from 15 seconds to 2 minutes longer than in the final version ([15], p. 382). The time limits were shortened because the ample time made the test too easy, with literate men who took both tests obtaining scores on the Army Beta that were inflated compared to their scores on the Army Alpha. As a result, the test was poor at discriminating properly among low scoring examinees—which was the examinee population it was designed for—and the scores from the two tests were not comparable with the longer time limits.^6^ The Army Beta’s creators needed to make the test more difficult, and shortening the time limit was the easiest way to do so ([15], p. 382). What the psychologists did not know at the time was that the shortened time limit may have increased the variation of examinees’ scores, and therefore increased the correlation between the test scores and other variables [34]. This is because mental processing speed was later shown to be an important component of intelligence [35,36], and it is theoretically plausible that requiring rapid responses increased the breadth of cognitive abilities that the Army Beta measured. Ironically, one of the aspects of the Army Beta that Gould criticized most may have improved the Army Beta.

### 3.4. Criticism of the Belief that the Army Beta Measures Intelligence

Gould’s [1] final criticism was against early psychologists’ belief that the Army Beta measured intelligence. Quoting Boring, (as reported by Kevles [23]), Gould [1] called the belief “preposterous” (p. 205). Gould also stated that test administration conditions “… made such a thorough mockery of the claim that recruits could have been in a frame of mind to record anything about their innate abilities” ([1] p. 205), and that it was “… ludicrous to believe that [the Army] Beta measured any internal state deserving the label intelligence” ([1], p. 210).

Kevles ([23], p. 573) also recorded Boring’s opinion in the 1960’s that “… the tests had predictive value …” because they correlated with meaningful outcomes in a soldier’s army career. Yet, Gould did not communicate this information to his readers. Additionally, Boring twice published articles in scholarly journals using the Army Alpha and/or Army Beta to measure intelligence [34,37]. In both articles, Boring accepts that the army tests measure intelligence.

Moreover, the degree to which the psychologists who designed the Army Beta believed that it measured “innate intelligence” is debatable. The massive 890-page report on the army testing program [15] uses the phrase “innate intelligence” only twice (on pp. 780 and 813), and neither passage stated that the army mental tests examined this construct. Never in the book did the authors make a strong argument that these tests could measure “innate abilities” ([1], p. 205). In fact, in one passage, Yerkes ([15], p. 811]) explicitly stated that a poor environment and bad health could lead to low test scores. 

But the issue is complicated by the committee authorship of the Yerkes [15] and Yoakum and Yerkes [16] books. It is difficult to find a consistent viewpoint of whether intelligence tests measure “innate” intelligence. On the one hand, many psychologists in the early 20th century did not believe that intelligence tests could tap into one’s “innate” abilities, free of environmental influence. As an example, one of the leading psychologists in the design of the Army Alpha and Army Beta, Lewis Terman, recognized before World War I that environmental influences could impact an examinee’s IQ score [28,38] and that intelligence could not be divorced from the influence of schooling [39]. Many other independent psychologists of the time did not believe that the tests only measured “innate ability” (e.g., [40,41]); the idea that intelligence tests were free from environmental influences was not an unquestioned viewpoint among American psychologists at the time ([5,42,43]. 

However, the Yoakum and Yerkes [16] report on the army mental testing program on the army mental tests is more favorable to Gould’s argument because it includes three passages that claim that the tests could measure “innate intelligence” ([16], pp. 8, 17, 192). Gould did not seem to consult this important source of information regarding the Army Alpha and Army Beta,^7^ though he did cite (in ([1], p. 199) a secondary source that quotes the Yoakum and Yerkes volume ([16], p. 17). 

The tangle of authorship and opinions in the Yerkes [15] and Yoakum and Yerkes [16] shows that it is far from clear that the army psychologists viewed their tests as measuring inborn intelligence that was immune to the influences of environment. Even when a specific passage of known authorship refers to the Army Alpha and/or Army Beta as measuring innate intelligence, it is not difficult to find quotations by the same author that also show a recognition that intelligence scores were influenced by environmental variables. For example, when discussing army mental performance of foreign-born men, Yerkes (the man, not the placeholder name for the group of army psychologists) endorsed the idea of meaningful differences in intelligence among immigrant groups in one article ([18], pp. 364–365). But in another article from the same year, when reviewing the same data, Yerkes cautions against ranking groups and mentions self-selection for immigration as a confound ([17], p. 242), leaving the interpretation ambiguous (cf. also Terman and Fernald in [16], p. 17; with [28], p. 135).

Additionally, the psychologists’ own data supported the argument that Army Alpha and Army Beta scores were affected by environmental influences, such as schooling. For example, Yerkes [15], p. 766) wrote that, “… subgroups which are successively better schooled make successively better showing in intelligence examinations.” The army intelligence test creators also recognized the importance of environment when they reported an entire chapter of data showing that immigrants who had lived in the United States longer scored higher on intelligence test scores than more recent immigrants ([15], pp. 701–704). Another chapter in Yerkes’s report is dedicated to examining the correlation between intelligence test scores and acquired physical health conditions (e.g., parasitic diseases, venereal disease), and found no major differences with healthy recruits. One may wonder why the army psychologists would even undertake such investigations if they all believed that their tests measured an innate ability that was unsullied by non-genetic influences. The most plausible reason is that at least some of the army psychologists recognized that the environment could have an impact on intelligence test scores, even when one of their goals in designing the Army Alpha and Army Beta was to minimize the influence of external sources on test scores as much as possible.

Gould ([1], pp. 217–222) admitted that Yerkes grappled with environmental influences, but Gould presented several examples where Yerkes apparently dismissed the possibility of environmental influences on intelligence test scores. However, Yerkes never stated that the test creators believed that correlational data disproved the hypothesis of environmental causes of differences in intelligence test scores. Most of the passages Gould quoted are embedded in larger sections of the Yerkes [15] report where it is clear that there is no conclusive causal evidence and in which Yerkes entertained multiple possible explanations for an observed correlation. Gould admitted this sometimes, but couched it in language that still portrayed Yerkes as having an unshakeable faith that intelligence test scores were not influenced by environmental variables. For example, when discussing Yerkes’s open admission that immigrants’ time in the United States was positively correlated with their test scores, Yerkes never gave a definite answer for why this correlation existed. (Indeed, doing so would not be supported by correlational data.) Instead, he considered multiple plausible reasons, including “successful” immigrants remaining in the country longer, and said that future research was needed. Gould stated this passage as indicating that “Yerkes admitted the possibility [of an environmental cause for the correlation], but held out strong hope for a hereditarian salvation …” (p. 221). While attitudes concerning heritability at the time did vary, the army psychologists avoided conclusive statements and tended to recognize potential limitations in their evidence.

Where the army psychologists did favor non-environmental explanations, this was after controlling for—as much as was possible, given the statistical and technological means available at the time—confounding variables. For example, Yerkes ([15], pp. 779–783) reported on the correlation between education level and intelligence test scores, and is quoted by Gould ([1], p. 218) as stating, “The theory that native intelligence is one of the most important conditioning factors in continuance in school is certainly borne out by this accumulation of data” ([15], p. 780). Setting aside the fact that this quote does not deny the potential importance of the environment on continuing one’s education, Gould did not state in his book that this quote comes amidst a report of correlations calculated among samples with homogeneous backgrounds (e.g., Black examinees in northern states, Black examinees in southern states, foreign-born White examinees, native-born illiterate examinees). Yerkes made these comparisons within these subsamples of examinees because he recognized that comparing groups with heterogeneous backgrounds would confound the relationship between variables, even if such comparisons would strengthen the correlations by reducing restriction of range ([15], pp. 779–780). Even after these adjustments, the correlations were still positive and noteworthy: the median correlations between total intelligence test scores and years of education reported in this section’s Tables 324 and 327 is *r* = 0.65 for the Army Alpha and 0.55 for the Army Beta. Given these data, it is not unreasonable to conclude that “… although an intelligent man may drop out of school at almost any stage beyond grade 3 an unintelligent man is most unlikely to remain in school beyond the eighth grade. Distinctly more than average intelligence would seem to be a prerequisite to a college education and almost as strictly a prerequisite to graduating from or even entering high school.” ([15], p. 783).

Gould also neglected much of the validity research in the two reports about the Army Beta. Although these studies are primitive by modern standards, they provide a great deal of evidence in favor of the belief that the Army Beta measured an examinee’s developed intelligence. Scores on the Army Beta correlated positively with scores on other intelligence tests, including the Army Alpha (*r* = 0.811) and the Stanford-Binet (*r* = 0.727), both the “gold standard” of intelligence measurement at the time ([15], p. 634). Army Beta scores also correlated positively with external criteria, such as the number of years of schooling an examinee had (for both children and adults), commanding officers’ ratings of soldiers’ job performance, and army rank [15,16]. Moreover, the Army Beta subtests all positively intercorrelated ([15], pp. 390, 634), and we demonstrate below that these correlations fit a one-factor model of intelligence. This model is consistent with modern mainstream theories of intelligence that incorporate a general reasoning factor or ability (e.g., [44,45]). All of this evidence not only supports the army psychologists’ beliefs about the manifestation of intelligence in the real world, but also is an example of the nomological net of correlations that one would expect if the Army Beta—or any other test—measured intelligence [46,47]. By the standards of the time, the amount of evidence was massive and exceeded the validity evidence for almost any other test. All that information was mutually supporting and consistent with the belief that the Army Beta measured intelligence. Gould [1] never once considered this validity information, and most of his readers would never know that this evidence existed.

## 4. Replication

### 4.1. Hypotheses

To determine the viability of Gould’s criticism of the Army Beta time limits and his claim that the Army Beta could not measure intelligence, we replicated his administration of the test to a sample of undergraduate students. We hypothesized that in an administration of the Army Beta to modern college students, we would find the following results:

1. Scores on the total Army Beta test would be as high or higher (α = 0.05) than Gould’s [1] students’ scores: 58% of students rated as A, 30% of students rated as B, and 11% of students rated as C. We believed this outcome was plausible for two reasons. First, Gould’s students were enrolled in a course on “biology as a social weapon” and may have had a preconceived bias against the utility and/or validity of the Army Beta; Gould’s treatment of Morton’s data and his negative view of the Army Beta’s creators may have also influenced the way he administered the Army Beta. Second, we thought it was plausible that the Flynn Effect would boost the scores of a modern sample of college students.
2. Completion rates of the new sample would be similar to completion rates in Gould’s [1] sample, as determined by a chi-squared test (α = 0.05). We created this hypothesis in order to test whether Gould’s results were consistent with an administration of the Army Beta according to the instructions ([15], pp. 162–165).
3. All variables in the correlation matrix would be positively correlated, and those correlations would be statistically significant (one-tailed test, α = 0.05), with the possible exception of Test 1’s score (which we believed would have low variance because it appeared to be an easy test and only allowed a maximum of 5 points out of 118). If this hypothesis were disproved, it would support Gould’s claim that the Army Beta could not measure intelligence (e.g., [1], p. 210). If Army Beta subscores all positively correlate, it would demonstrate that the Army Beta functions much like any other intelligence test.
4. Confirmatory factor analysis would show good fit for a one-factor congeneric model. This one-factor model would fit the data better than the two-factor model that would consist of two correlated factors, where one factor consisted of subtests that required written numbers (Cube Analysis, Digit Symbol, and Number Checking) and a second factor consisted of all other subtests (Maze, X-O Series, Picture Completion, and Geometric Construction). This two-factor model is consistent with Gould’s claim that the use of written numbers could interfere with the test’s ability to measure intelligence, whereas a one-factor model would be consistent with the Army Beta’s creators’ view that the test measured a general cognitive ability, and that it was rational to combine the subtest scores into a global test score. Thus, Hypothesis 4 pits our interpretation of the Army Alpha and Gould’s interpretation against one another to determine which is better supported by the data.

It is important to note the different purposes that the hypotheses serve. The first two are related to how the Army Beta functions in modern samples; Hypothesis 1 examines Army Beta scores in two different university contexts, and Hypothesis 2 serves as a check on the integrity of Gould’s [1] administration of the Army Beta. The other two hypotheses are more relevant to the psychometric validity of interpreting Army Beta scores as a measure of intelligence. Finding evidence in support of Hypotheses 3 and 4 would disprove Gould’s [1] claim that the Army Beta was incapable of measuring intelligence.

### 4.2. Methods

Participants in our replication were a convenience sample—like Gould’s, though our students were enrolled in an introductory psychology course at our institution (an open enrollment university). Students took the Army Beta during their normal class time in a large classroom, and they received course credit for their participation. The study received ethical approval from the university’s Institutional Review Board. A total of 210 introductory psychology students participated in our study, of whom there were 205 who completed all subtests and for whom data are reported in this article. Of the examinees, 91 (44.4%) were male, 111 (54.1%) were female, and 3 (1.5%) chose not to report their sex. The majority of the examinees were White (183, 89.3%), with smaller numbers who reported that they were Hispanic (30, 14.6%), “Other” (9, 4.4%), Asian (7, 3.4%), Native American (7, 3.4%), Black (6, 2.9%), or Pacific Islander (4, 2.0%). Race/ethnicity totals do not sum to 205 because participants were instructed to select all race/ethnicity categories that applied to them. The average Army Beta score was 88.7 (SD = 9.96), which is the equivalent of a C+, or “high average,” rating for a World War I draftee. The self-reported GPAs of our sample were high (M = 3.50, SD = 0.54), while the ACT scores were consistent with the university’s open enrollment mission (M = 23.1, SD = 3.8). Using the equation provided by Koenig et al. (2008, p. 157), these ACT scores are the equivalent of a mean IQ of 103.6 (SD = 2.58).^8^

Gould indicated that he followed the instructions from Yerkes ([15], pp. 162–165), which are extremely detailed. To facilitate administration and to be transparent in our methods, we made a video of the administration instructions, which is available at https://www.youtube.com/watch?v=HAMQPJyUiuo. This video replicates Yerkes’s instructions with a few minor exceptions. First, on the Cube Analysis subtest, the real Army Beta instructors had a small model that they showed examinees to convey that the picture represented a three-dimensional object. We omitted the model because we could not find suitable cubes that didn’t have symbols on them, and we preferred plain cubes because we believed that symbols might be distracting. Gould ([1], p. 205) indicated that he used baby blocks for this purpose.) Second, on the Geometric Construction subtest, the World War I examiners demonstrated the first three example problems with paper cutouts. In the video, we only used paper cutouts for the first example problem because our demonstration cutouts were not to the same scale as the visual aid on the wall. Other differences between our video and the administration of the Army Beta in the late 1910’s include the modern civilian dress of the individuals in the video, the multimedia (instead of live) presentation format, and the fact that the demonstration occurs on white pieces of paper using dark markers, instead of on a dark chalkboard using white chalk. Of these differences, we believe that only the first two were major deviations from the original administration protocols. Even these deviations likely did not create problems for our sample because the vast majority of examinees still understood what to do, as indicated by the large numbers who attempted the items on the subtests.

Replication study participants received a packet that consisted of the informed consent document, the Army Beta test, and a short survey asking about the students’ gender, racial/ethnic heritage, college admissions test scores, and overall college grade-point average (GPA). The informed consent document stated that examinees were taking the Army Beta in order to help psychologists to better understand the test. Demographic data, college admissions test scores, and college GPA data were not reported in Gould’s [1] book, but we asked these questions so that we could report basic demographic information about our sample and also gather validity evidence via correlations between Army Beta total scores and self-reported ACT and self-reported college (GPA) scores. The self-reported ACT scores served as a modern measure of intelligence. The ACT is a college admissions test that contains four sections (English, Reading, Mathematics, and Science) and is based on the typical American high school curriculum in these topics (see [48] for evidence that the ACT is a test of intelligence). The self-reported college GPA was chosen to be a measure of real-world achievement and success in the students’ environment. All study materials are available on the Open Science Framework at http://osf.io/y6njt.

To test Hypothesis 1, we conducted a chi-squared test to examine whether the distributions of A, B, or C ratings in Gould’s sample and the replication sample were similar. A chi-squared test was also used to test Hypothesis 2, though in this case, the completion rates of sample members for each subtest were compared. We tested Hypothesis 3 with a series of Pearson’s *r* correlations. Finally, Hypothesis 4 was tested with the specified confirmatory factor analysis models. All analyses were pre-registered (with an exception noted in Table 2) at https://osf.io/y6njt/ after data were collected and cleaned but before any analyses occurred. We pre-registered our analyses because we recognized that we had a positive bias towards the Army Beta’s creators’ work and a pre-existing negative view of *The Mismeasure of Man*. As a result, we wanted to minimize the flexibility we had in the analysis and minimize the chance that our results would be tailored to support our pre-existing beliefs. We invite readers to compare the time-stamped pre-registration of our analyses with the results reported in this document and verify that our analyses matched our original data analysis plan.

### 4.3. Results

In regards to Hypothesis 1, Table 2 shows the number of college students in each sample who earned Army Beta ratings of A, B, or C. A chi-squared test indicated that Gould’s students earned disproportionately more A ratings and fewer C ratings than the replication sample (χ^2^ = 52.671, df = 2, *p* < 0.001). This disproved our hypothesis that the replication sample would perform as well or better than Gould’s due to the impact of the Flynn Effect on our sample or Gould engaging in prejudicial test administration.

Hypothesis 2 was supported by our data, which is displayed in Table 1. Of the seven Army Beta subtests, two of them (Number Checking and Geometric Construction) were finished by similar proportions of both samples (*p* ≥ 0.210). Of the remaining five subtests, two (Maze and Digit Symbol) were completed by more replication sample members, while three (Cube Analysis, X-O Series, and Picture Completion) were completed at higher rates of Gould’s sample members (all *p* values < 0.05). Thus, there is no consistency in the completion rates of the Army Beta subtests for the different samples.

Hypothesis 3 was mostly supported. Table 3 shows the correlation among the Army Beta subtest and total test scores. As predicted, the correlations among Army Beta scores were generally positive. However, three were not statistically significant (those equal to or below *r* = 0.111). Contrary to our expectations, only one of these involved the Maze subtest, whereas the other two were correlations of the X-O Series subtest with other Army Beta subtests. There was also one negative correlation (*r* = −0.015) between the Picture Completion subtest score and college GPA, which was evidence against our hypothesis, though this correlation did not statistically differ from zero (exploratory analysis two-tailed *p* = 0.848). These low correlations may be due to the low reliability for most subtests (Cronbach’s α values between 0.349 and 0.737). Despite the low reliability of the overall Army Beta scores (α = 0.489), total scores still positively correlated with self-reported ACT Composite scores (*r* = 0.379, *p* < 0.001) and self-reported college GPA (*r* = 0.143, *p* = 0.033), which we predicted in our pre-registration.

Finally, Hypothesis 4 stated that the Army Beta data would fit a one-factor model, instead of a plausible alternate two-factor model. Table 4 shows the fit statistics for both models in the replication sample. Statistically, the model fit was equal for both models (Δχ^2^ = 2.040, df = 1, *p* = 0.153), and the other fit statistics were nearly identical. Given the equally good fit for both models, the scientific principle of parsimony would favor the one-factor model over the two-factor model. This view is strengthened by the high correlation between the two factors (*r* = 0.842).

To further test the viability of the one-factor model, we performed confirmatory factor analysis on two correlation matrices of Army Beta data for World War I draftees ([15], pp. 390, 634). These (non-pre-registered) statistical tests are also reported in Table 4. Inevitably, the two-factor model had a lower χ^2^ value than the one-factor model, though only one of these comparisons was statistically significant. (This was for the largest sample’s Δχ^2^ comparison, which is unsurprising, given the Δχ^2^ test’s extreme sensitivity to sample size.) However, this is likely because the two-factor model is slightly less constrained than the one-factor model, and the χ^2^ test favors more complex models. A comparison of the other fit statistics (RMSEA, CFI, TLI, SRMR) shows that each sample had nearly identical fit statistics across both models, and where the two-factor model was a better fit, it was almost inevitably for fit statistics that do not penalize complex models (χ^2^, CFI, and SRMR). Thus, even in two samples of World War I draftees—groups that would be much more likely to have a separate factor for recognition of written numbers than a modern sample of college students—a one-factor model fit the data just as well as a two-factor model. Again, parsimony dictates that researchers should favor a one-factor model of intelligence from the historic Army Beta data. These results supported our hypothesis and the confirmatory factor analysis data in our replication sample. The decision to favor a one-factor model is also supported by the extremely high correlation between the two factors (*r* = 0.975 in both samples), which makes it highly unlikely that the two factors are conceptually different, and provides internal validity evidence for the belief that the Army Beta measures a general cognitive ability in all three samples. 

Table 5 shows the standardized factor loadings for all models and the correlation between factors (for two-factor models). This table also supports the argument that a one-factor model fits better for our sample, as demonstrated by the higher factor loadings for the one-factor model. Likewise, for the historical Army Beta samples, Table 5 shows that factor loadings are extremely similar in one- and two-factor models, which is unsurprising, given the extremely high correlation between factors (meaning that these factors are almost identical). Thus, data from all three samples in Table 5 also show that a one-factor model either fits better (for our sample) or is more parsimonious (for the World War I samples) than a two-factor model. This means that the Army Beta likely measures a single general cognitive ability.

It is worth noting that in our sample, the factor loadings in our replication sample were lower in the two-factor model than in the one-factor model, sometimes substantially, so (e.g., X-O series factor loading in the one-factor model is 0.940, but in the two-factor model the loading is only 0.244). This is a phenomenon which did not occur in the two Yerkes [15] samples (see Table 5). This likely shows that—for our more educated sample—the distinction between subtests that require writing numbers and subtests that do not is artificial. The lower factor loadings indicate that the subtests in the two-factor models were lower quality measures of the theorized underlying factor, even though each factor had fewer observed variables as indicators. This indicates that the factors in the two-factor model were not coherent groupings of observed variables. In the Yerkes [15] samples, however, the high correlation between factors in the two-factor model (*r* = 0.975) meant that the two factors were essentially the same, which is why the factor loadings changed very little from the one-factor model to the two-factor model. It also indicates that Gould’s [1] distinction between subtests that required writing numbers and other subtests was not meaningful.

### 4.4. Discussion

Given these results from our replication, it seems that Gould’s criticism of time limits and his argument that the Army Beta did not measure intelligence are without basis. Despite the short time limits for each Army Beta subtest, the results of this replication support the World War I psychologists’ belief that the Army Beta measured intelligence. We demonstrated this in the following four relevant results of our replication:

First, Harvard students scored higher on the test than students at an open-enrollment university. This contradicts our hypothesis; we believed that a Flynn Effect would inflate a modern sample’s scores on an intelligence test compared to an early 1980’s sample. We either overestimated the impact of the Flynn Effect on Army Beta scores or underestimated the discrepancy between the cognitive abilities of our sample and Gould’s students. In our retrospective analysis, it seems that the latter possibility was very powerful in explaining why Gould’s students performed better than our sample. We were not able to find early 1980’s mean college admissions test scores for Harvard University, but incoming freshmen in 1991 at Harvard averaged 1390 on the SAT [49], which is the equivalent of a composite score 30 on the ACT. This is about 7 points higher than our sample’s mean self-reported ACT score. Given the current ACT standard deviation of 5.22 reported in the ACT’s technical manual, our modern sample scores 1.3 standard deviations lower on the ACT than early 1990’s Harvard students. It seems unlikely that a Flynn Effect (which usually averaged 0.2 to 0.33 standard deviations per decade in the United States during the 20th century) could have caused our sample to equal or exceed the performance of Gould’s on the Army Beta. While this seems obvious in retrospect, it was not obvious to us when we collected the data. 

Second, completion rates for Gould’s [1] sample and the replication sample were statistically equal for two subtests, higher on three subtests for Gould’s sample, and higher on two subtests for the replication sample. We believe this shows that Gould probably did administer the Army Beta to his students in an honest manner, despite his opinions about the test.

Additionally, the subtest scores on the Army Beta almost all correlate positively with one another—just as one would expect from the positive manifold (though 2 of the 21 correlations were statistically equal to zero). Moreover, the total Army Beta scores positively correlate with two external criteria that are commonly used to validate intelligence tests: academic achievement (in the form of self-reported college GPA) and scores from another intelligence test (i.e., self-reported ACT composite scores).^9^ This is precisely the nomological network of findings that one would expect from a test of intelligence and positive evidence that the Army Beta measures skills and/or abilities that are useful on succeeding in a 21st century academic context. It is important, though, to recognize that both of these external criteria were self-reported, and it is a limitation of our study that neither of these variables was verified for accuracy. Additionally, because of the open enrollment nature of our institution, some sample members may have never taken a college admissions test at all.

Finally, internal validity evidence was demonstrated when a one-factor model fit the data from three separate samples, two of which were World War I draftees. A plausible two-factor model based on Gould’s [1] claim that the understanding of written numbers would interfere with the measurement of intelligence in illiterate men also fits the data, but no better than a one-factor model. Thus, the more parsimonious model (i.e., the one-factor model) is preferred. It is unlikely that the use of written numbers interferes with the Army Beta’s ability to measure nonverbal intelligence, either in a modern sample or in an illiterate sample from a century earlier. Additionally, the existence of a coherent factor supports the test creators’ belief that the Army Beta subtests could combine into a measure of general cognitive ability.

Any one piece of presented evidence regarding the validity of the belief that the Army Beta measured intelligence is, by itself, not very compelling. However, the combination of all the results from our empirical examination of the Army Beta—the higher scores for students at a prestigious college, the positive correlations with external criteria (another test of cognitive ability and with academic achievement in our replication), and the coherent general factor that emerges from three independent datasets—provides mutually reinforcing evidence that the test measures general intelligence as defined by mainstream psychologists (e.g., [44,45]) to an extent. Additionally, Yerkes [15] and Yoakum and Yerkes [16] reported positive correlations between army test scores and other external criteria, including supervisor ratings, civilian job prestige, military rank, and years of education. While each of these pieces of evidence does not conclusively disprove Gould’s contention that it is “… ludicrous to believe that Beta measured any internal state deserving the label intelligence” ([1], p. 210), the weight of evidence we present is against Gould. We believe that the test as a whole functions much like theorists—from the early 20th century or early 21st century—would expect for a test of intelligence. Had the correlation matrices better supported the two-factor model, or if Army Beta scores had correlated negatively with college academic performance or ACT scores, we would have found some support for Gould’s view that the Army Beta did not measure intelligence. Instead, we find that the Army Beta functions much as other intelligence tests do.

## 5. Overall Discussion

### 5.1. Gould’s Judgments of the Army Beta

Among the many topics of negative analysis in Stephen Jay Gould’s *The Mismeasure of Man* [1] is the Army Beta test. Although not the most prominent section of Gould’s text, his 23-page passage on the Army Beta is typical of his style in the book. Throughout the book, Gould criticized early scientists who studied individual and group differences of being misled by preconceived notions based on their social beliefs—instead of the data. Yet, Gould himself was motivated to write *The Mismeasure of Man* by his strong political and social beliefs, which guided him to present his text describing the early intelligence scientists as blinded by their prejudices [4,7,12,50]. Given Gould’s pervasively incorrect statements in *The Mismeasure of Man* about the Army Beta, factor analysis [3], the place of intelligence testing in the immigration debates of the 1920s [5,9,10], the biological basis for intelligence [4,8,9], and the questions regarding Gould’s analysis of Morton’s work [11,12,13,14], we wonder whether there is *any* section of *The Mismeasure of Man* that is factually accurate.

Like other sections of *The Mismeasure of Man*, when Gould wrote about the Army Beta, he omitted relevant information that contradicted his preconceived beliefs and misinterpreted data in order to portray the study of individual human differences as ideological pseudoscience. Contrary to Gould’s claims, the Army Beta’s content, instructions, and time limits were all appropriate for a group-administered intelligence test a hundred years ago. We believe we have also demonstrated that the Army Beta very likely measured intelligence, given the results of multiple confirmatory factor analyses and the positive correlations with external criteria (both during World War I and in modern times). 

The completion data from Gould’s replication, detailed in Table 1, indicates that he likely did make a sincere effort to administer the Army Beta according to the test’s original instructions ([15], pp. 162–165). Many of Gould’s criticisms (e.g., language ability, cultural awareness, or the innateness of measured intelligence) are generally relevant to discussions about measuring intelligence. However, Gould’s interpretation of the Army Beta and the historical record tends to mischaracterize and misrepresent early psychometricians and the value of the Army Beta. Instead, we demonstrated that the test really did measure intelligence—and still does to some extent in a modern sample (although with low reliability for many subtests). 

### 5.2. Other Thoughts

Although not the principal aim of our study of the historical record and our replication, we had realizations during the course of our work on this article that we think are relevant to understanding the Army Beta. First, we garnered a great deal of respect for the army psychologists as we scrutinized their reports about the Army Alpha and Army Beta [15,16] and as we created the Army Beta administration video. These psychologists were early practitioners of open science, and their descriptions and instructions are so detailed that we found little ambiguity in their reports about how the tests were created, piloted, refined, administered, scored, or used. Almost every question we had about the tests, the examinee population, or the development process was answered explicitly in the Yerkes [15] and/or Yoakum and Yerkes [16] books. Their example inspired us to also embrace open science practices and make all material, data, analyses, and computer code available to readers at https://osf.io/y6njt/ through the Open Science Framework web site. 

We were also impressed by the level of sophistication of the tests. When one considers that group intelligence testing was in its infancy and that there was almost no scholarly research about how to design instructions, choose time limits, or select item formats, the creation—in less than a year—of two sophisticated tests that were then administered to over 1 million men is an incredible accomplishment. The Army Beta is especially remarkable in this regard because nonverbal testing was largely uncharted territory for this first generation of test creators. Only three Army Beta subtests had clear precursors in the scholarly literature: the Maze subtest [51], the Digit Symbol subtest [52], and the Picture Completion subtest [53]. Two subtests had conceptual forerunners, but were still largely original in format and stimuli: the Number Checking subtest (which Yerkes [15], p. 369), claimed was based on a previous test from Thorndike, but this test seems to have remained unpublished) and the Geometrical Construction subtest (based on form boards, which date to the 1800’s; see [43], p. 13). The two remaining subtests (Cube Analysis and X-O Series) were entirely new. We find it impressive that the army psychologists created multiple novel subtests and so many original stimuli for the Army Beta in such a short period of time.

We were also pleased that the Army Beta can still function as a test of intelligence 100 years after it was created. Although the confirmatory factor analysis results show that the intelligence factor in the modern sample is weaker than the general factor that emerged from the World War I data (possibly due to the low reliability of the subtest scores and/or restriction of range in our modern sample), a one-factor model of the Army Beta still fits the data well. This leads us to believe that the Army Beta can still be used in research purposes for measuring intelligence. There are some advantages to this test: researchers can administer the test quickly, and the use of our video adds to the ease and consistency of administration. The Army Beta requires no verbal responses, and many of its subtests require little specific cultural knowledge beyond knowing how to use a pencil or understanding written numbers (though the Picture Completion test is an exception in this regard). Additionally, the Army Beta is in the public domain, which means that researchers can use it free of charge. One final advantage is that the subtests (and the test as a whole) were subject to decades of research, and there are often cross-cultural data available about how these subtests function with non-English speaking populations (e.g., [54]). However, the test is not suitable for high-stakes assessment, and there are shortcomings in the Army Beta when compared to modern tests—especially in regards to the reliability of the scores in our replication sample. An interesting future study would be to administer the test to a group with an education and/or English familiarity level closer to that of a typical World War I examinee than our college-educated sample was.

## 6. Conclusions

Given the knowledge, technology, and test development standards of the time, the Army Beta was an adequate test for its specific purpose. Indeed, the data from our replication indicate that the test may be a suitable measure of intelligence even for a modern sample, despite any prejudices or ulterior motives that may have existed among the test creators at the time. We believe that Stephen Jay Gould [1] was largely incorrect in his assessments of the Army Beta and that his approach to the Army Beta is largely representative of *The Mismeasure of Man* as a whole (see [55,56] for other perspectives of how Gould distorted research topics that he finds socially distasteful). The test’s content, instructions, and time limits were appropriate for the examinee population at the time. Additionally, our replication data—and the information that the army psychologists provided about the test’s creation, administration, and validity evidence—all point to the same conclusion: that the Army Beta measured intelligence, and can still somewhat do so 100 years later.

## Figures and Tables

**Table 1:** Completion Counts for Army Beta Subtests in Gould’s [1] Sample and the Replication Sample.

		Gould’s Sample		Replication Sample			
Subtest #	Subtest Name	Completed	Not Completed	Completed	Not Completed	χ^2^ (*p*)	Odds Ratio ^a^
1	Maze	44	9	191	14	5.345	2.791
		(83.0%)	(17.0%)	(93.2%)	(6.8%)	(0.021)	
2	Cube Analysis	21	32	44	161	7.368	0.416
		(39.6%)	(60.4%)	(21.5%)	(78.5%)	(0.007)	
3	X-O Series	45	8	131	74	8.568	0.315
		(84.9%)	(15.1%)	(63.9%)	(36.1%)	(0.003)	
4	Digit Symbol	12	41	99	106	11.304	3.191
		(22.6%)	(77.4%)	(48.3%)	(51.7%)	(0.001)	
5	Number Checking	18	35	52	153	1.574	0.661
		(34.0%)	(66.0%)	(25.4%)	(74.6%)	(0.210)	
6	Picture Completion	49	4	150	55	8.877	0.223
		(92.5%)	(7.5%)	(73.2%)	(26.8%)	(0.003)	
7	Geometric Construction	40	13	157	48	0.029	1.063
		(75.5%)	(24.5%)	(76.6%)	(23.4%)	(0.865)	

^a^ Reference group is Gould’s sample and reference outcome is not completing the subtest. Therefore, OR values greater than 1 indicate that the replication sample was more likely to complete the subtest. OR values less than 1 indicate that Gould’s sample was more likely to complete the subtest.

**Table 2:** Army Beta Intelligence Ratings for Gould’s Sample [1] and the Replication Sample.

Rating	Gould’s Sample	Replication Sample
A	31 (58.5%)	29 (14.1%)
B	16 (30.2%)	65 (31.7%)
C	6 (11.3%)	111 ^a^ (54.1%)

*Note*. χ^2^ = 52.671 (df = 2, *p* < 0.001). This particular chi-squared test was not pre-registered because of an oversight in the pre-registration process. However, we did pre-register a hypothesis that the replication sample would score as well or better than Gould’s sample. This statistical significance test was conducted to test this hypothesis, which was disproved, as indicated by the larger proportion of replication sample members who earned scores in the C range and the smaller proportion who earned scores in the A range. ^a^ In the replication sample, 68 examinees received a rating of C+, 38 examinees received a rating of C, and 5 examinees received a rating of C−.

**Table 3:** Correlation Matrix of Army Beta Subtests Scores and Total Score (*n* = 205).

	Maze	Cube Analysis	X-O Series	Digit Symbol	Number Checking	Picture Completion	Geometric Construction	Total Score	ACT Composite ^a^	College GPA ^b^
**Maze**	1.000									
**Cube Analysis**	0.229	1.000								
**X-O Series**	0.134	0.160	1.000							
**Digit Symbol**	0.157	0.273	0.045	1.000						
**Number Checking**	0.224	0.286	0.152	0.292	1.000					
**Picture Completion**	0.111	0.274	0.127	0.106	0.128	1.000				
**Geometric Construction**	0.236	0.313	0.093	0.229	0.231	0.319	1.000			
**Total Score**	0.361	0.638	0.372	0.638	0.709	0.486	0.558	1.000		
**ACT Composite Score ^a^**	0.114	0.280	0.185	0.060	0.270	0.315	0.294	0.379	1.000	
**College GPA ^b^**	0.061	0.135	0.193	0.094	0.084	−0.015	0.001	0.143	0.104	1.000
**Cronbach’s α**	0.349	0.619	0.737	0.698	0667	0.366	0.563	0.489	—	—

^a^*n* = 143. ^b^ *n* = 165.

**Table 4:** Confirmatory Factor Analysis Results for Army Beta Data.

Sample	Model	χ^2^ (df, *p*)	Δ χ^2^ (df, *p*)	RMSEA [90% CI]	CFI	TLI	SRMR
Replication	1 factor	13.139	—	0.000 [0.000, 0.064]	1.000	1.011	0.034
(*n* = 205)		(df = 14, *p* = 0.516)					
Replication	2 factors	11.099	2.040	0.000 [0.000, 0.060]	1.000	1.026	0.032
(*n* = 205)		(df = 13, *p* = 0.603)	(df = 1, *p* = 0.153)				
Yerkes ([15], p. 390)	1 factor	104.446	—	0.099 [0.082, 0.118]	0.965	0.948	0.032
(*n* = 693)		(df = 14, *p* < 0.001)					
Yerkes ([15], p. 390)	2 factors	106.581	2.135	0.102 [0.085, 0.120]	0.966	0.945	0.033
(*n* = 693)		(df = 13, *p* < 0.001)	(df = 1, *p* = 0.144)				
Yerkes ([15], p. 634)	1 factor	226.542	—	0.117 [0.104, 0.131]	0.950	0.925	0.038
(*n* = 1102)		(df = 14, *p* < 0.001)					
Yerkes ([15], p. 634)	2 factors	221.567	4.975	0.121 [0.107, 0.135]	0.951	0.921	0.039
(*n* = 1102)		(df = 13, *p* < 0.001)	(df = 1, *p* = 0.026)				

*Note*. The two-factor models all consisted of two correlated factors, the first of which consisted of the subtests that required the use of written numbers (Cube Analysis, Digit Symbol, and Number Checking). The second factor consisted of the remaining subtests. The factors were allowed to correlate, and no subtest cross-loaded onto another factor. All error terms were kept uncorrelated with one another. All models were identified by setting factor variances to 1.

**Table 5:** Standardized Factor Loadings, Factor Correlation, and Percentage of Variance Explained by Factors for Army Beta Data.

Subtest Name	Replication Sample		Yerkes ([15] p. 390)		Yerkes ([15], p. 634)	
	1 Factor	2 Factors	1 Factor	2 Factors	1 Factor	2 Factors
Maze	0.842	0.403	0.622	0.630	0.607	0.614
Cube Analysis	0.636	0.623	0.726	0.719	0.722	0.712
X-O Series	0.940	0.244	0.816	0.821	0.812	0.814
Digit Symbol	0.813	0.456	0.859	0.869	0.842	0.855
Number Checking	0.766	0.501	0.821	0.832	0.794	0.807
Picture Completion	0.832	0.444	0.754	0.758	0.755	0.759
Geometric Construction	0.698	0.601	0.714	0.721	0.723	0.727
*n*	205	205	693	693	1102	1102
Factor Correlation	—	*r* = 0.842	—	*r* = 0.975	—	*r* = 0.975

*Note*. The two-factor models all consisted of two correlated factors, the first of which consisted of the subtests that required the use of written numbers (Cube Analysis, Digit Symbol, and Number Checking). The second factor consisted of the remaining subtests. The factors were allowed to correlate, and no subtest cross-loaded onto another factor. All error terms were kept uncorrelated with one another. All models were identified by setting factor variances to 1.

## Footnotes

1 The omitted portion of the quotation is three pages of history describing Gould’s and his family’s social advocacy. It is clear in this passage that he saw *The Mismeasure of Man* as being a continuation of this tradition of political work.

2 The text of this section in revised version of *The Mismeasure of Man* [7] is unchanged from the original edition of the book, so all references will be to the 1981 edition.

3 The phonograph item was the third most difficult item in the Picture Completion subtest for our replication sample. Only 32.7% of examinees who attempted it answered the question correctly, though Items 8 (a picture of an envelope) and 10 (a picture of a pocketknife), were more difficult for our examinees.

4 About 8% of Army Beta examinees were born in the UK or Canada ([15], p. 696, Table 213), and it is reasonable to believe that most of these spoke English as a native language. It is unclear, though, whether immigrants from these countries were disproportionately more likely to be recent immigrants.

5 Gould [1] never reported the percentage of zero scores on each subtest, only that these were excessive. The only table stating exact zero percentages for each subtest ([15], p. 741) showed that between 2.1% and 26.9% of White examinees and 4.5% to 36.0% of Black examinees earned a zero on each Army Beta subtest. These numbers are not representative of the entire examinee population, though, because they are from a single location, Camp Dix, New Jersey. In a comparison of fifteen military training camps, Camp Dix had the second-lowest mean Army Beta score (based on data from [15], p. 669). It is not clear why Camp Dix scored lower than others; Shuey ([32], pp. 314–315) believed it was because almost half of the examinees at Camp Dix were Black or foreign-born Whites, many of whom would be more likely to be poorly educated than literate, native-born White men. Garrett ([33], p. 484) also identified different procedures for selecting examinees for Army Beta that could have caused the lower scores at Camp Dix.

6 The army psychologists’ attempts to make scores on both tests interchangeable may be the earliest example of test score equating in the scientific literature.

7 This would be consistent with some past criticisms of Gould’s book. Snyderman and Herrnstein [5] and Carroll [3] also criticized Gould’s knowledge of the history of intelligence testing.

8 The “high average” rating is for all examinees across the Army Alpha and Army Beta. The mean score on Beta for a sample of 26,012 examinees ([15], p. 669, Table 189), was about 40.45 (SD = 21.50), which indicated that our sample scored 2.24 SD above the mean for illiterate soldiers in World War I. A total Army Beta score of 40.45 warranted a rating of D, or “inferior”.

9 When correcting for low reliability of the total Army Beta test score and the restriction of range for our examinees, the correlations increased to *r* = 0.812 for self-reported ACT scores and *r* = 0.411 for self-reported college GPA. It is interesting that the former correlation is almost exactly equal to the *r* = 0.811 correlation between Army Beta scores and total Army Alpha scores that Yerkes ([15], p. 634) reported. However, because these corrections were not pre-registered we are relegating them to this footnote instead of the main text of the article. Readers should put more stock in our pre-registered analyses.
